# Plant-Growth Synchronized, Acid Phosphatase-Responsive Lignin-Based Controlled Release Phosphorus Nanofertilizers

**DOI:** 10.1021/acs.biomac.5c02594

**Published:** 2026-04-27

**Authors:** Alice Boarino, Nicola Carrara, Joaquin Clua, Nick Zahnd, Yves Poirier, Harm-Anton Klok

**Affiliations:** † Institut des Matériaux and Institut des Sciences et Ingénierie Chimiques, Laboratoire des Polymères, 27218 École Polytechnique Fédérale de Lausanne (EPFL), Station 12, Lausanne 1015, Switzerland; ‡ Department of Plant Molecular Biology, 54403 University of Lausanne, Lausanne 1015, Switzerland; § Instituto de Biología Molecular y Celular de Plantas, Consejo Superior de Investigaciones Científicas, Universitat Politècnica de València, Valencia 46011, Spain

## Abstract

Deploying agrochemicals as nanoparticle-based formulations not only provides opportunities to tune release kinetics, prevent premature degradation, increase shelf life, and reduce loss of active ingredient but also can enable the design of systems that can interact with or respond to soil and/or plants in a specific manner, which provides further opportunities to refine the delivery of agrochemicals. This article presents tripolyphosphate (TPP)-cross-linked lignin-based nanofertilizers that are designed to disintegrate and release phosphorus upon exposure to acid phosphatase, which is an enzyme that is upregulated as part of the phosphate starvation response of plants. In model experiments, it was shown that phosphorus release from the lignin-TPP nanoparticles was triggered by acid phosphatase, dependent on the enzymatic activity, and accompanied by the simultaneous disintegration of the nanoparticles. Experiments with the model plant *Arabidopsis thaliana* showed that lignin-TPP nanoparticles are an efficient phosphorus source for plants, suppressing the typical growth inhibition and activation of the molecular mechanisms triggered by phosphate deficiency. These experiments underline the potential of lignin-TPP nanoparticles in providing readily accessible phosphate for plants during growth and development, which represents a step forward toward nanofertilizers that are able to release their payload on demand in a plant-growth-synchronized manner.

## Graphical abstract

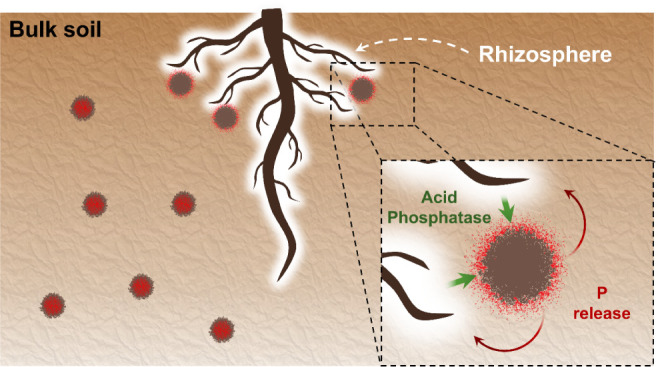

## Introduction

The world population is projected to grow to nearly 9.8 billion by 2050.^1,2^ To secure global access to food, this will require a 50% increase in agricultural productivity by 2050 as compared to 2012.^3,4^ While the use of agrochemicals and new technologies has been essential to meet the demand for food for the growing world population over the past century,^5^ current agricultural practices are not efficient and unsustainable.^6^ As an example, from the more than 150 million tons of fertilizer that are globally applied every year, only 30–50% of nitrogen and 18–20% of phosphorus reach the target crop.^7–9^ The inefficient use of agrochemicals also contributes to greenhouse gas emissions and global energy consumption^1,5,7,10^ and overburdens nonrenewable natural resources such as phosphate rock, which is critical for the production of phosphorus fertilizers.^11,12^

To promote a more efficient and sustainable use of fertilizers, there has been an increasing interest in the development of controlled or slow release fertilizers, which can help to prevent premature degradation, increase shelf life, and reduce the loss of active ingredients due to leaching and evaporation.^13–15^ One possible and promising approach toward controlled release fertilizers involves the use of nanoparticle-based formulations.^8,16–25^ Nanoparticles can be designed to interact with or respond to soil or plants in a specific manner, which provides further opportunities to refine the delivery of agrochemicals. Examples of controlled release nanofertilizers include organic, polymer, and inorganic (e.g., calcium phosphate and hydroxyapatite-based formulations).^13,20,26–29^ Release of fertilizer from these nanoparticles depends on and can be tuned by varying nanoparticle composition and can be facilitated by leveraging variations in environmental parameters such as temperature, pH, redox potential, ionic strength, as well as soil microbial activity.

In addition to abiotic stimuli such as pH and temperature and enzymes produced by, for example, plant pathogens, another attractive strategy to trigger and tune release from nanofertilizers would be to exploit enzymes that are released by plant roots during development and growth. This would be attractive, as it may ultimately allow access to nanofertilizers that would release their payload in a plant-growth-synchronized manner. One example of a process that may provide an avenue toward plant-growth-synchronized nanofertilizers is the starvation-induced secretion of acid phosphatase,^30,31^ which is an enzyme that plays a key role in plant phosphorus metabolism.^32^ Plants uptake phosphorus as soluble inorganic orthophosphate via their root system. Under phosphate-deficient conditions, plants initiate a variety of processes, which are collectively termed the phosphate starvation response. One of these responses includes the induction and secretion of acid phosphatase enzymes into the rhizosphere (i.e., the narrow region of soil that is affected by plant roots), which can hydrolyze phosphate ester bonds from a plethora of substrates.^33^ Designing nanofertilizers that release their payload in response to root acid phosphatase may allow for nutrient delivery that is driven by the needs of the plant.

This study demonstrates the potential of tripolyphosphate (TPP)-cross-linked aminated lignin nanoparticles to provide on-demand, acid phosphatase-driven release of phosphorus for plant development. These nanofertilizers are based on aminated lignin, which has been explored as a slow/controlled release fertilizer.^34^ The amine groups allow the incorporation of TPP cross-links. These cross-links provide acid phosphatase responsiveness and serve to trigger disintegration of the nanoparticles, and simultaneous conversion of TPP into orthophosphate, which is then released and available as a plant nutrient.^35^ The changes in nanoparticle size, as well as the concomitant release of phosphorus, were investigated in the presence of different enzyme concentrations by dynamic light scattering, atomic force microscopy, and the malachite green assay. The ability of these nanofertilizers to release phosphate to sustain plant growth was tested on *A. thaliana* assessing the plant shoot weight, phosphate content, and the activation of the phosphate starvation response at the molecular level.

## Results and Discussion

The acid phosphatase-responsive nanofertilizers explored in this study were generated from aminated lignin and TPP, as illustrated in Scheme 1. The preparation of the nanoparticles starts with the ionic gelation of aminated lignin with TPP, followed by covalent cross-linking using *N*-(3-(dimethylamino)­propyl)-*N*′-ethylcarbodiimide (EDC) as a coupling agent. For the preparation of the nanofertilizers, lignin was used since it has been widely employed as a coating for conventional nitrogen- and phosphorus-based fertilizers (e.g., urea, triple superphosphate).^13,36–40^ Lignin biodegrades in soil^41^ and is characterized by a high carbon content, which can increase the organic matter in soil and have a beneficial influence on soil and plant health, acting as a biostimulant.^36,37^ Lignin is an attractive material for agricultural applications, as it is available in huge quantities in the form of lignocellulosic biomass that is generated as a side product of the paper and bioethanol industry. For the preparation of the nanofertilizers investigated in this study, aminated lignin was used, which was obtained by successive phenolation and Mannich modification of soda lignin (Supporting Information Scheme S1). The soda lignin had a number-average molecular weight (*M*_n_) of 2000 g/mol and a dispersity (Đ) of 1.80, as determined by gel permeation chromatography (GPC). Phenolation and Mannich modification did not result in significant changes in GPC molecular weight and dispersity (see Supporting Information Table S1). Elemental analysis of the Mannich-modified lignin revealed a nitrogen content of 8.5%, as compared to 0.5% for the soda lignin starting material (Supporting Information Table S1). The nitrogen content found in the soda lignin starting material is in agreement with that reported in other studies.^42^ The presence of these amine groups allows for reaction with TPP and the formation of cross-linked lignin/TPP nanoparticles. The TPP cross-links also provide acid phosphatase responsiveness, which enables to trigger disintegration of the nanoparticles, and simultaneous conversion of TPP into orthophosphate, which is then released and available as a plant nutrient.

**Scheme 1:**
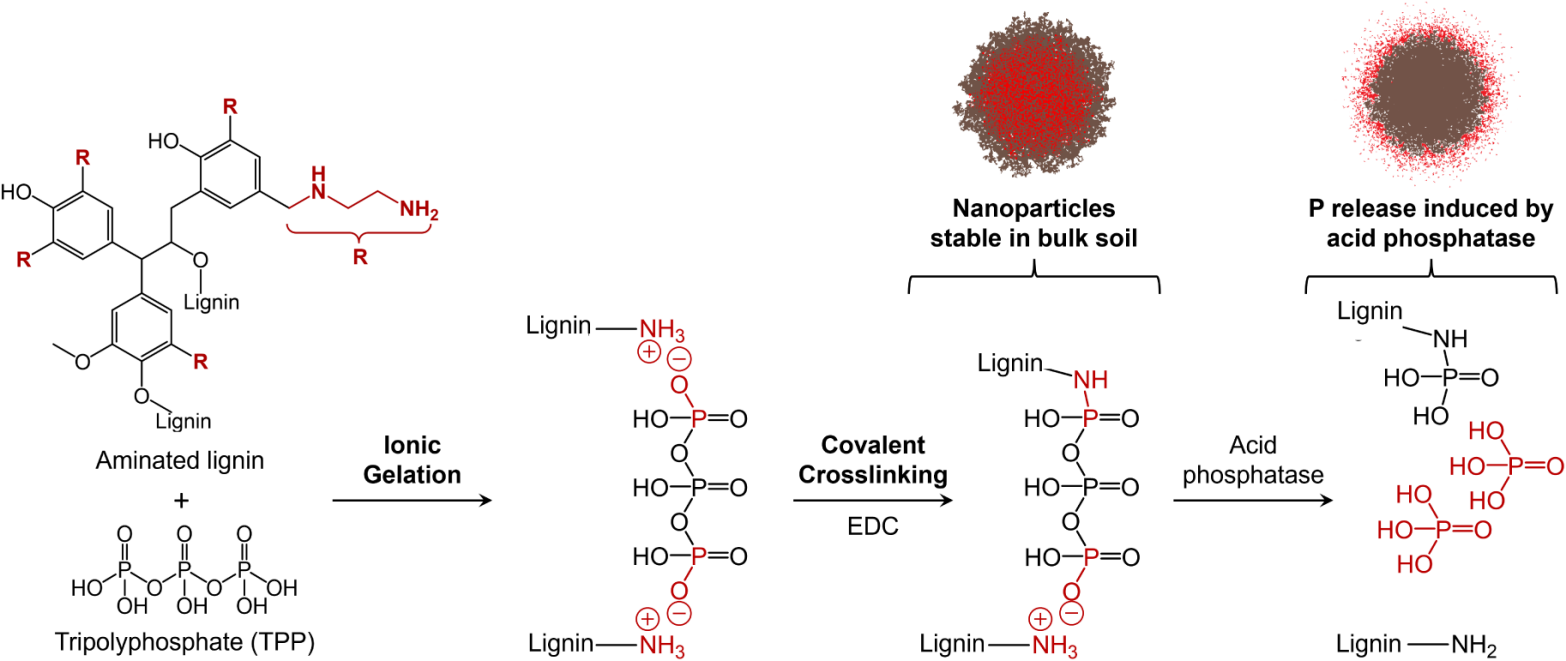
Preparation of Lignin/TPP Controlled Release Phosphorus Nanofertilizers^a^

a The chemical structure of the aminated lignin that is shown is a representative, idealized structural unit of this complex biopolymer.

### Preparation of Aminated Lignin/TPP Nanoparticles

The preparation of the lignin/TPP nanoparticles starts with the addition of TPP to a pH 2 aqueous solution of aminated lignin at an N/P molar ratio of 1.25. This results in the formation of ionically cross-linked particles with a diameter of 1021 ± 120 nm, a polydispersity index (PDI) of 0.381 ± 0.102, and a zeta potential of 2.4 ± 1.1 mV, as determined by dynamic light scattering (DLS) (Supporting Information Figure S1). The relatively large PDI, together with the near-neutral zeta potential, which may facilitate aggregation, indicates that the sizes that were determined do not necessarily reflect those of single particles but could represent particle aggregates. Subsequent addition of EDC to generate covalent phosphoramide cross-links resulted in smaller-sized and more uniform nanoparticles. DLS analysis of the EDC cross-linked nanoparticles in Milli-Q water indicated a size of 184 ± 1 nm, a PDI of 0.103 ± 0.011, and a zeta potential of 21.9 ± 2.1 mV (Figure 1A). In 2-(*N*-morpholino)­ethanesulfonic acid (MES) buffer at pH 5.7, which resembles the conditions that are used to grow the *Arabidopsis thaliana* plants for the experiments that will be presented below, DLS analyses revealed a particle size of 203 ± 3 nm, a PDI of 0.118 ± 0.015, and a zeta potential of 14.3 ± 3.3 mV (Figure 1A). Figure 1B presents an SEM image of covalently cross-linked nanoparticles that were first dispersed in water and then deposited and dried on a silicon substrate. Analysis of the particle size distribution indicates an average diameter of 42.3 ± 6.3 nm. This is smaller as compared to the results of DLS, which is due to the fact that DLS studies are performed in aqueous media, where the nanoparticles will swell, while scanning electron microscopy (SEM) analyzes the particle size and particle size distribution in the dry state. The cross-linked lignin/TPP nanoparticles have an N content of 6.3 wt % as determined by elemental analysis, while inductively coupled plasma mass spectrometry (ICP-MS) analysis results in a P content of 11.13 wt % (Supporting Information Table S1). These results correspond to a molar ratio of N:P of 1.25, which agrees with the 1.25:1 N:P ratio that was used to prepare the nanoparticles and with the positive zeta potential of the cross-linked lignin/TPP nanoparticles.

**Figure 1:**
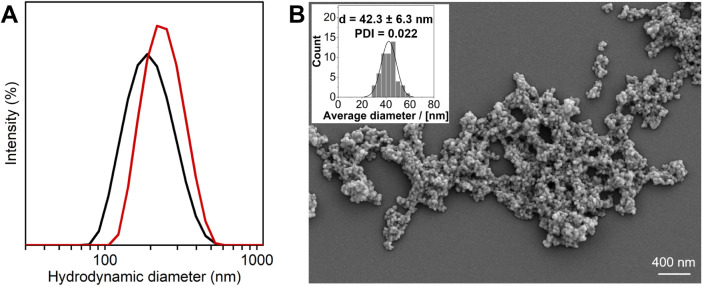
**A)** DLS curves of covalently cross-linked lignin/TPP nanoparticles dispersed in Milli-Q water (black line) and MES buffer 2.5 mM at pH 5.7 (red line). **B)** SEM image and size distribution of covalently cross-linked lignin/TPP nanoparticles after dispersion in Milli-Q water, deposition, and drying on a silicon substrate.

The lignin/TPP nanoparticles were further characterized by Fourier-transform infrared (FTIR) spectroscopy and X-ray photoelectron spectroscopy (XPS). Figure 2 compares the FTIR spectra of TPP and aminated lignin with those of the ionically and covalently cross-linked lignin nanoparticles. The FTIR spectrum of the covalently cross-linked nanoparticles displays signals at 1260, 980, and 760 cm^–1^, which are consistent with the formation of phosphoramide bonds.^43–45^ The presence of the two bands at 1640 and 1600 cm^–1^ in the spectrum of the covalently cross-linked nanoparticles, which are due to the amine and protonated amine groups, indicates that not all lignin amine groups are converted upon reaction with TPP in the presence of EDC into phosphoramide bonds. All further experiments described in this paper were performed on the covalently cross-linked lignin nanoparticles.

**Figure 2:**
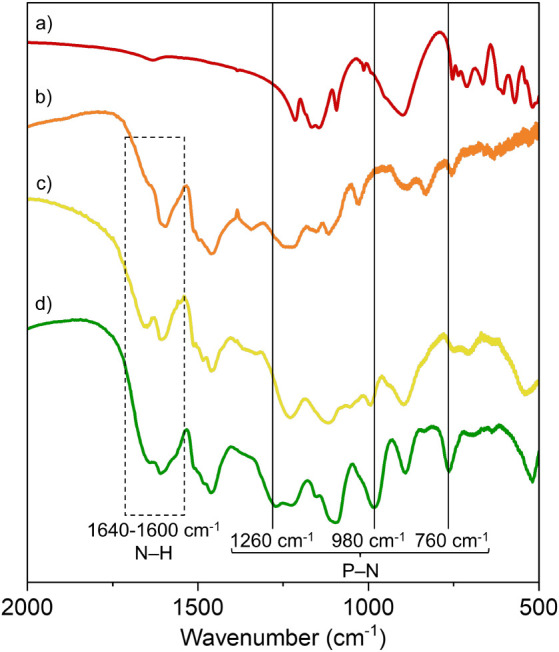
FTIR spectra of a) TPP, b) aminated lignin, c) ionically, and d) covalently cross-linked nanoparticles.

Figure 3 presents N 1s high-resolution XPS scans of aminated lignin and ionically and covalently cross-linked nanoparticles. The N 1s spectrum of the aminated lignin can be fitted with 2 residuals: at 401.5 eV for the protonated amino groups (−NH_3_^+^) and at 399.6 eV for the nonprotonated primary amine (−NH_2_) and secondary amine groups (−NR_2_).^46–49^ Comparison of the N 1s spectrum of the aminated lignin with that of the ionically cross-linked particles reveals an increase in the intensity of the component at 401.5 eV, which is consistent with the formation of ionically cross-linked lignin/TPP particles. The N 1s XPS spectrum of the covalently cross-linked lignin/TPP nanoparticles shows an increase in the intensity of the component at 399.6 eV relative to that at 401.5 eV. These changes are consistent with the formation of covalent, phosphoramide bonds and indicate that the final lignin/TPP nanoparticles incorporate both ionic as well as covalent cross-links.

**Figure 3:**
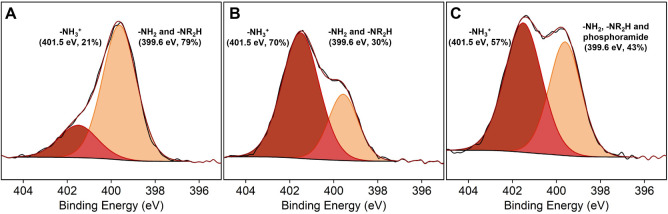
N 1s XPS high-resolution scans of **A)** aminated lignin, **B)** ionically, and **C)** covalently cross-linked nanoparticles. R = H, alkyl group.

### Acid Phosphatase-Triggered Phosphate Release

The release of phosphorus from the covalently cross-linked lignin/TPP nanoparticles in response to acid phosphatase was first evaluated in model experiments in pH 5.7 MES buffer. MES buffer was selected to resemble the conditions that are used for the plant experiments that will be presented later. For these experiments, 0.2 mg/mL nanoparticles were dispersed in MES buffer in the presence of different units of enzyme activity per volume inside a dialysis cassette, and the release of free phosphate into the dialyzate monitored with the malachite green assay over a period of 80 h (Figure 4A). The enzyme-responsiveness of the nanoparticles was evaluated at acid phosphatase activities of 0.1, 1, and 10 mU/mL to resemble the concentration of this enzyme in the rhizosphere.^50^ Figure 4B presents the evolution of the phosphate concentration in the dialyzate over time. In the absence of the enzyme (0 mU/mL), no phosphate was released. In the presence of the enzyme, in contrast, a gradual increase in the concentration of free phosphate was observed. The rate of phosphorus release was proportional to the unit activity of acid phosphatase per volume. For experiments that used 1 and 10 mU/mL acid phosphatase, after ∼50 h, a plateau concentration of ∼14 μM was reached, which corresponds to 65 wt % of the total phosphorus in the lignin/TPP nanoparticles. The results in Figure 4 demonstrate the retention of phosphorus in the absence of the enzyme and an acid phosphatase-triggered, activity-dependent release of phosphorus in the presence of the enzyme, which highlights the potential of the lignin/TPP nanoparticles as controlled release nanofertilizers.

**Figure 4:**
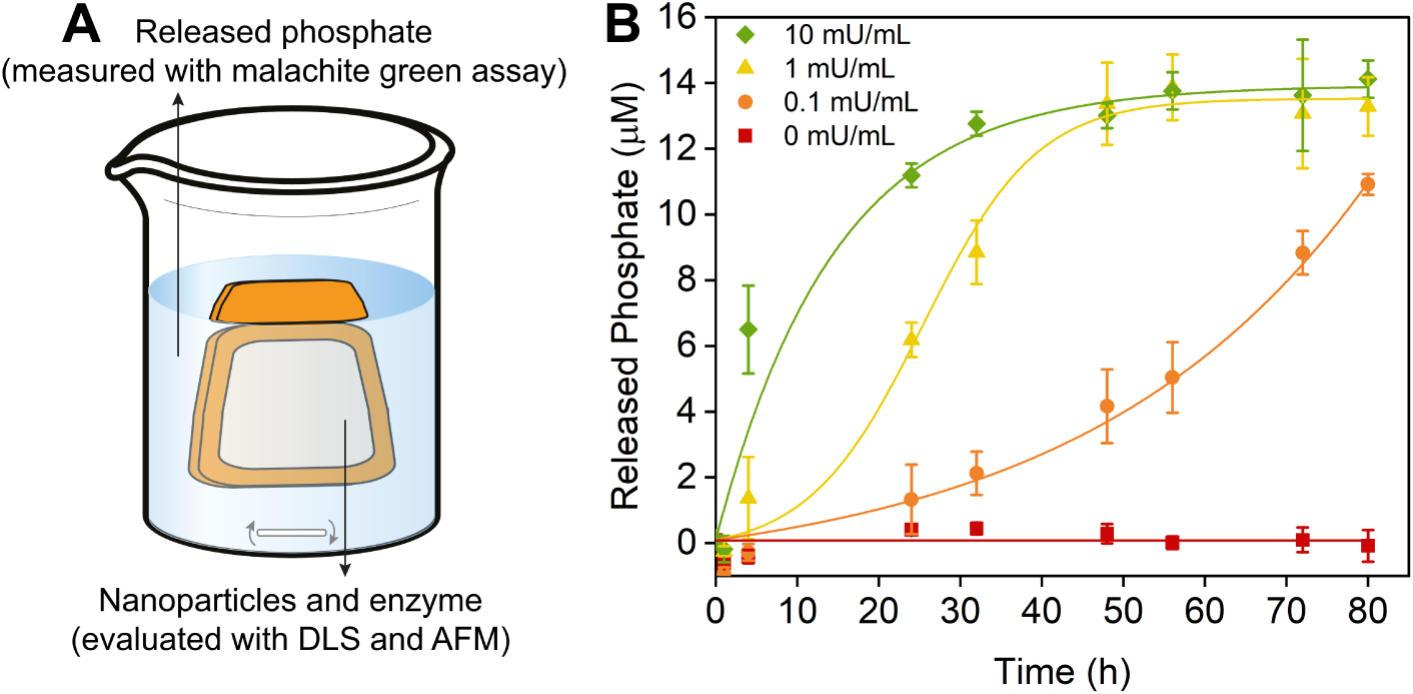
**A)** Schematic illustration of the experiment that was used to evaluate the enzyme-triggered release of phosphate from the nanoparticles. **B)** Concentration of phosphate released in the dialyzate over time at acid phosphatase activities of 10, 1, 0.1, and 0 mU/mL, quantified by the malachite green assay.

To study the impact of acid phosphatase-mediated phosphorus release on the nanoparticles, the retentate of the dialysis experiment (Figure 4A) was studied by DLS. Figure 5 presents the size distribution of lignin/TPP nanoparticles over a period of 72 h, both in the absence of acid phosphatase as well as upon exposure to 1, 10, and 50 mU/mL enzyme. In the absence of acid phosphatase, no noticeable changes in the size distribution are observed. Also, the zeta potential of the particles remains constant at ∼10 mV over 72 h (Supporting Information Figure S2). In the presence of 10 and 50 mU/mL acid phosphatase, the size distribution shifts to larger diameters and becomes multimodal. This reflects a continuous decrease in the cross-link density of the particles as phosphorus is released (and a concomitant swelling and increase in particle diameter), ultimately leading to the disintegration of the nanoparticles. These changes in particle size distribution are accompanied by a gradual increase in the zeta potential to >20 mV. These observations are consistent with the gradual degradation of the nanoparticles and the release of phosphorus. Analysis of the size distribution of TPP/lignin nanoparticles exposed to 1 mU/mL acid phosphatase reveals similar changes in size distribution, albeit less pronounced, which is consistent with the reduced rate of phosphorus release under these conditions that was observed with the malachite green assay (see Figure 4B).

**Figure 5:**
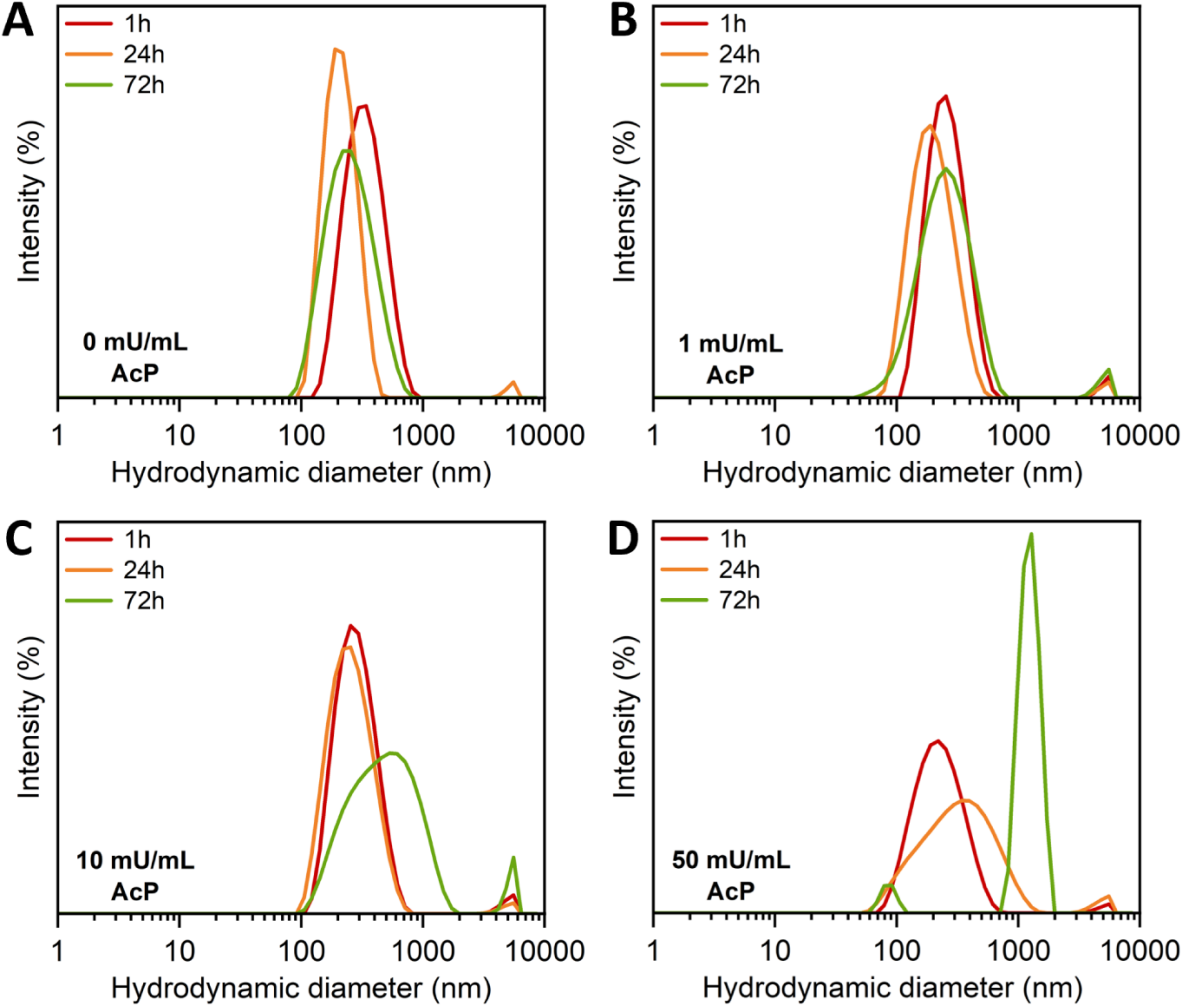
Particle size distribution of nanoparticles incubated with A) 0 mU/mL, B) 1 mU/mL, C) 10 mU/mL, and D) 50 mU/mL acid phosphatase (AcP), measured at different time points (1, 24, and 72 h) with DLS.

The degradation of the covalently cross-linked lignin/TPP particles upon exposure to acid phosphatase was also monitored by atomic force microscopy (AFM). Figure 6 presents a series of images that was obtained from nanoparticles that were exposed to 10 mU/mL acid phosphatase. In Figure 6A, which was acquired at time 0, the nanoparticles have a spherical shape. In Figure 6B, C, and D, recorded after 24, 48, and 72 h of incubation with the enzyme, respectively, a gradual change in morphology and aggregation into larger clusters with undefined shapes is observed, which is consistent with the enzyme-mediated degradation of the nanoparticles.

**Figure 6:**
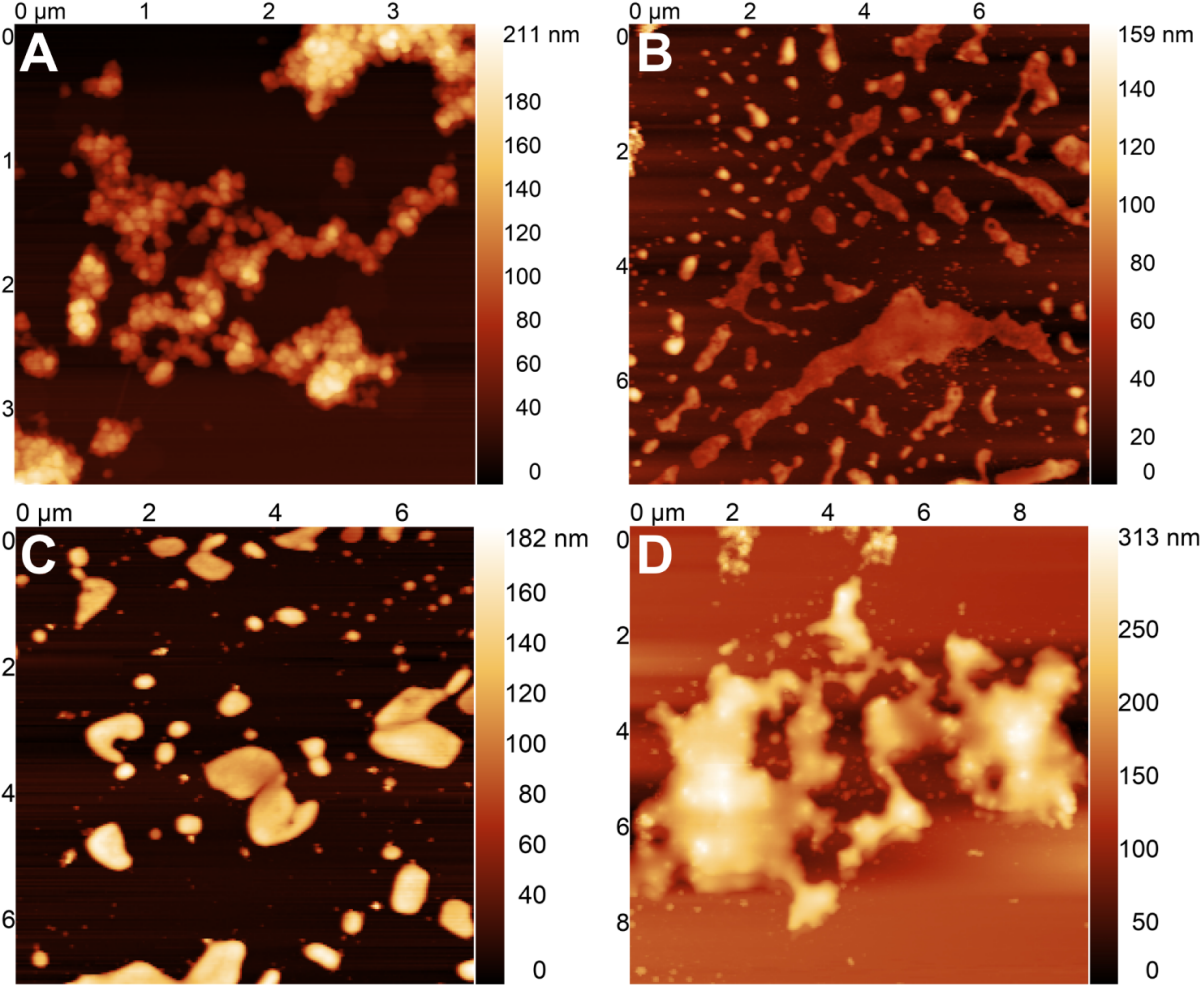
AFM images of cross-linked lignin/TPP nanoparticles after **A)** 0 h, **B)** 24 h, **C)** 48 h, and **D)** 72 h of incubation with acid phosphatase (10 mU/mL).

### Evaluation of Lignin/TPP Nanoparticles to Support *A. thaliana* Growth under Phosphate Deficiency

To assess their performance as controlled release phosphorus nanofertilizers, the ability of the cross-linked lignin/TPP nanoparticles to sustain the growth of *A. thaliana* was studied. *A. thaliana* was selected as a model plant since its physiological and molecular responses toward phosphate deficiency are well described.^51–54^ Numerous aspects of the adaptation of plants to phosphate deficiency are shared between *A. thaliana* and crop plants, making this model plant an appropriate subject to test lignin/TPP nanoparticles as a source of phosphate.^55–58^ For these experiments, *A. thaliana* was grown for 4 weeks in a clay-based substrate with a phosphate-free medium. Plants were irrigated once per week with a phosphate-free mineral solution (0 mM P Buffer) or the same solution supplemented with either different concentrations of P buffer (1 mM P Buffer or 0.5 mM P Buffer) or an equivalent amount of phosphate-containing nanoparticles (1 or 0.5 mM P nanoparticles). As a control, plants were irrigated with solutions containing lignin nanoparticles that did not incorporate TPP (no P nanoparticles). To assess the ability of the lignin/TPP nanoparticles to deliver phosphate, plant growth and development were monitored by determining shoot weight and soluble shoot phosphate content, as well as by analyzing the expression of the canonical phosphate starvation response marker gene monogalactosyldiacylglycerol synthase 3 (MGD3), induced by phosphate starvation 1 (IPS1) and SYG1/Pho81/XPR1 domain-containing protein 1 (SPX1).^33^ Figure 7 presents photographs taken from plants that were grown under these different conditions, as well as the results of the analysis of phosphate content and phosphate starvation marker gene analysis.

**Figure 7:**
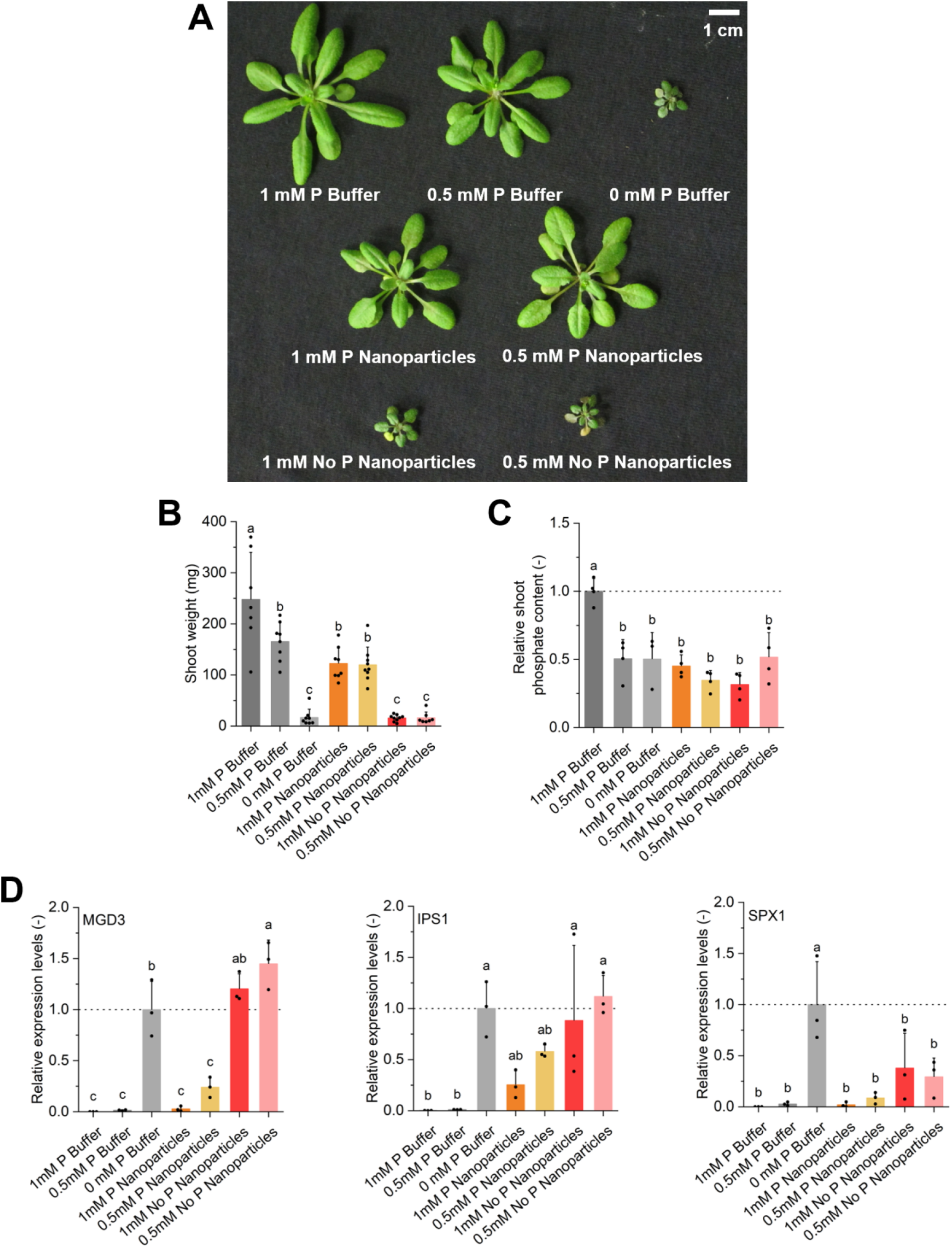
**A)** Images of representative shoots from *A. thaliana* plants irrigated with a mineral solution without phosphate (0 mM P Buffer) or the same media supplemented with the indicated concentrations of phosphate buffer (0.5 mM P Buffer, 1 mM P Buffer), phosphate-containing nanoparticles (P Nanoparticles), or nanoparticles without phosphate (No P Nanoparticles). Plants were grown for 4 weeks in a clay-based substrate, and representative shoots were excised and photographed. **B)** Fresh shoot weight of plants grown as aforementioned. **C)** Soluble P quantification from shoots plotted as P content relative to the 1 mM P Buffer control. **D)** Relative expression levels of the canonical P starvation responsive genes *MGD3*, *IPS1*, and *SPX1* normalized to the 0 mM P Buffer condition. In **B**, **C,** and **D,** graphs present the mean and standard deviation. Replicates are indicated by black dots. Statistically significant differences were assessed by a one-way ANOVA followed by Tukey’s test with a *p*-value <0.05. Significant differences between samples are indicated by different letters.

Plants grown without a phosphate source (i.e., no lignin/TPP nanoparticles and 0 mM P) show a strong inhibition of shoot development as compared to plants that were provided with 1 or 0.5 mM P (Figure 7A and B). Application of 1 mM P buffer translated into higher biomass production and shoot phosphate content as compared with plants that were grown using 0.5 mM P buffer (Figure 7B, C). As plants grown with 0.5 mM P buffer have the same shoot phosphate content as those that were kept with 0 mM P buffer, this suggests that the 1 mM P buffer condition provides an excess of phosphate, which the plant uses for biomass production and storage in the vacuole, whereas 0.5 mM P supplies just enough phosphate to sustain normal plant growth without excess storage (Figure 7C).

As illustrated by the photographs in Figure 7A, deploying cross-linked lignin/TPP nanoparticles that contain an equivalent of 0.5 or 1 mM P allows to support growth and development of *A. thaliana*. At 0.5 mM P lignin/TPP nanoparticles, there are no significant differences in shoot weight and phosphorus content as compared with the free buffer control experiment at the same P concentration (Figure 7B and C). Increasing the dose of lignin/TPP nanoparticles to 1 mM, however, did not result in a concomitant dose-dependent increase in shoot weight and shoot phosphate content as was observed for the experiments with the P buffer.

To understand the effect of lignin/TPP nanoparticles on the plant phosphate starvation response, the relative expression levels of canonical marker genes were analyzed (Figure 7D). The results revealed that plants treated with 0.5 and 1.0 mM lignin/TPP nanoparticles do not show a significant induction of MGD3 or SPX1 expression levels compared to the use of 1 mM P or 0.5 mM P buffer, suggesting that plants are not experiencing P deficiency at the molecular level. On the other hand, the expression levels of IPS1 are intermediate between those of P deficiency conditions (i.e., 0 mM P) and P sufficiency (i.e., 1 mM P). This could reflect a cellular status where plants just take up enough P to sustain growth when lignin/TPP nanoparticles are provided, given that the hydrolysis of the TPP bonds requires enhanced synthesis and secretion of acid phosphatases, as compared to plants that are provided with free phosphate in solution. This could also explain why the soluble phosphate content of 1 mM P nanoparticle-treated plants is similar to those treated with 0.5 mM P nanoparticles, as well as the mild induction of MGD3 and SPX1 (albeit not statistically significant) compared to the 1 and 0.5 mM P conditions. These observations highlight the potential of lignin/TPP nanoparticles as promising phosphorus controlled release nanofertilizers that are able to synchronize phosphate release with the metabolic needs of the plant without further depleting the P content of the soil.

## Conclusions

Nanoparticles that are able to release plant nutrients in response to changes in specific soil biochemical parameters may provide a way to reduce the premature release of these actives, help reduce their environmental impact, and increase the efficiency of the use of these agrochemicals. This study has demonstrated that the incorporation of phosphorus in the form of TPP in lignin-based nanoparticles provides nanofertilizers that are responsive to acid phosphatase, which is an enzyme that is secreted by plants as part of the phosphate starvation response. In model experiments, it was shown that P release from the lignin/TPP nanoparticles was triggered by acid phosphatase, dependent on the enzymatic activity, and accompanied by the disintegration of the nanoparticles. Experiments with *A. thaliana* that were grown under phosphate-deficient conditions indicated that lignin/TPP nanoparticles suppress the phosphate starvation response and are able to sustain plant growth and development. These experiments illustrate the potential of enzymes secreted by plants into the rhizosphere to trigger on-demand nutrient release. This represents a step forward for nanofertilizers that can release their payload in a plant growth-synchronized manner. Ultimately, this may help to mitigate the negative environmental impact and allow for the more efficient use of agrochemicals.

## Experimental Section

### Materials

Soda lignin (Protobind 1000, *M*_n_ = 2000 g/mol, *M*_w_ = 3800 g/mol, and Đ = 1.80 as measured by GPC) was purchased from Tanovis AG, Switzerland. Phenol, 37% aqueous formaldehyde solution, sulfuric acid (H_2_SO_4_), hydrochloric acid (HCl), ethylenediamine, *N*-(3-(dimethylamino)­propyl)-*N*′-ethylcarbodiimide (EDC), deuterated dimethyl sulfoxide (DMSO-d6), sodium tripolyphosphate (TPP), 2-chloro-4,4,5,5-tetramethyl-1,3,2-dioxaphospholane (TMDP), acid phosphatase from wheat germ (activity >0.4 U/(mg solid) measured at pH 4.8 at 37 °C), 2-(*N*-morpholino)-ethanesulfonic acid (MES) with low moisture content, sodium citrate dihydrate, citric acid, *p*-nitrophenyl phosphate disodium salt hexahydrate (pNPP), and malachite green phosphate assay kit were supplied by Sigma-Aldrich. Deionized water was obtained from a Millipore Direct-Q 5 ultrapure water system. The agar (Micro agar) was purchased from Duchefa Biochemie, and the Murashige and Skoog (MS) medium without phosphate was supplied by Caisson Laboratories.

### Methods

#### Nuclear Magnetic Resonance (NMR) Spectroscopy

For ^1^H NMR spectroscopy, samples were dissolved in DMSO-d6 at a concentration of 10 mg/mL. Spectra were recorded on a Bruker AVANCE III 400 MHz spectrometer, and chemical shifts are reported relative to the residual solvent signal.

For ^31^P NMR spectroscopy, a 20 mg sample was precisely weighed and dissolved by stirring overnight at room temperature in 400 μL of a 0.8:0.8:1 v/v mixture of deuterated CDCl_3_, DMF-d7 and anhydrous pyridine, which contained 4 mg (0.0115 mmol) of chromium­(III)­acetylacetonate as a relaxation agent, and 8.21 mg (0.082 mmol) of cyclohexanol as an internal standard. Then, the lignin hydroxyl and carboxylic acid groups were phosphorylated by adding an excess (100 μL) of 2-chloro-4,4,5,5-tetramethyl-1,3,2-dioxaphospholane (TMDP). The reaction took only a few minutes to be completed,^59^ and the spectra were recorded the same day on a Bruker AVANCE III 400 MHz spectrometer.

#### Elemental Analysis

The C, H, and N % content in soda lignin, phenolated lignin, aminated lignin, and aminated lignin/TPP nanoparticles was determined in triplicate using a UNICUBE organic elemental analyzer (Elementar, Germany). The reported values are the averages of three measurements performed on each of three different samples.

#### Gel Permeation Chromatography (GPC)

GPC analysis was performed on a SECcurity^2^ GPC system (PSS Polymer Standards Service GmbH, Germany), equipped with a SECcurity^2^ refractive index detector, a GRAM precolumn of 50 mm length, and three GRAM columns of 300 mm length. All columns had a diameter of 8 mm and a particle size of 10 μm. Sample analysis was performed in dimethylacetamide (DMAc) + 0.9 wt % LiCl at 70 °C at a flow rate of 1.0 mL/min, using poly­(methyl methacrylate) (PMMA) standards.

#### Inductively Coupled Plasma Mass Spectrometry (ICP-MS)

To measure the phosphorus content in the nanoparticles, a 5 mg sample was subjected to acid digestion using 2 mL of concentrated HNO_3_ (69%, ROTIPURAN Supra, Roth) in polypropylene digestion vials using a heating block system (DigiPREP Jr., Fifteen mL, 40 Pos, SCP Science). Digestion was performed by first heating the sample to 100 °C in 15 min and subsequently maintaining the sample at this temperature for 60 min. After the digestion, the sample volumes were precisely adjusted to 5 mL with Milli-Q water. Samples were further diluted 300 times with 2% HNO_3_ solution, and their P content was analyzed by ICP-MS using KED mode with He as a collision gas on a NexIon 350 D ICP-MS instrument (PerkinElmer). Y­(NO_3_)_3_ was added as an internal standard at a concentration of 2 ppb to all the solutions, and P quantitation was performed using an external calibration curve with multielement standards (TraceCERT, 33 elements, 10 mg/L in nitric acid) in the 0.05–100 ppb range. All measurements were performed in triplicate.

#### Dynamic Light Scattering (DLS)

Average particle sizes (which are reported as intensity-average size distribution), polydispersity indices (PDI), and Z potentials were measured by dynamic light scattering using a Zetasizer Nano ZS (Malvern Panalytical Ltd, Spectris plc) instrument. For these analyses, the nanoparticles were diluted in Milli-Q water or in 2.5 mM MES buffer at pH 5.7 to a concentration of 0.05 mg/mL. For both Milli-Q and MES buffer, the refractive index and viscosity of water (1.330 and 0.8872 cP, respectively) were used. All measurements were taken after 10 min of sonication and with the attenuation value set between 7 and 9.

#### Scanning Electron Microscopy (SEM)

SEM images were acquired on a Zeiss Gemini SEM 300 at 3.00 kV. The samples were prepared by depositing a 4 μL drop of a diluted nanoparticle solution (0.05 mg/mL) on a silicon wafer and air-drying overnight at room temperature. A 10 nm protective layer of Au/Pd was applied before analysis on a Q150T Plus turbomolecular pumper (Quorum Technologies).

#### Atomic Force Microscopy (AFM)

An Asylum Research Cypher VRS instrument (Oxford Instruments, United Kingdom) was utilized for the acquisition of the AFM images. Measurements were done in tapping mode using an aluminum-coated silicon cantilever (POINTPROBE NCSTR-50, Nanoworld Technologies, Switzerland) with a spring constant of 7.4 N/m and a resonance frequency of ∼160 kHz. To prepare the samples, 4 μL of nanoparticle dispersion in Milli-Q water (to analyze particle sizes) or in 2.5 mM pH 5.7 MES buffer (to study the effect of acid phosphatase on the stability of the nanoparticles) at a concentration of 0.05 mg/mL was deposited on a silicon wafer, dried overnight at room temperature, and finally imaged to determine nanoparticle sizes and size distributions. The silicon wafers (10 mm × 8 mm in size) used for the experiments were previously cleaned via sonication in methanol, deionized water, and acetone for 10 min each. Images were processed with Gwyddion software. Particle sizes are reported as an average of 50 nanoparticle heights.

#### Fourier Transform Infrared (FTIR) Spectroscopy

FTIR spectra were acquired on a Nicolet 6700 instrument (Thermo Fisher Scientific) in the wavenumber range 500 to 4000 cm^–1^, using an average of 32 scans.

#### X-ray Photoelectron Spectroscopy (XPS)

Samples for XPS analysis were prepared by dissolving ionically cross-linked nanoparticles and dispersing covalently cross-linked nanoparticles at a concentration of 20 mg/mL in dioxane. After 2 h, the solutions were spin-coated onto 10 mm × 8 mm silicon wafers, which had been cleaned via 10 min sonication in methanol, deionized water, and acetone prior to use. Spin-coating was performed using a Convac ST 146 spin coater at 2000 rpm for 50 s. The samples were then dried under vacuum at 40 °C overnight to remove residual solvent. XPS measurements were carried out on an Axis Supra instrument (Kratos Analytical) using the monochromated Kα X-ray line of an aluminum anode. The pass energy was set to 20 eV with a step size of 0.1 eV. The samples were insulated, and an electron flood gun was used to limit the charging effect. Spectra were referenced at 284.8 eV using the aliphatic C–C bond of the C 1s orbital.

#### *Arabidopsis thaliana* Plant Growth

*Arabidopsis thaliana* Columbia-0 (Col-0) was grown for a period of 4 weeks on a clay-based substrate (Seramis) with phosphate-free 1/6 MS medium (Caisson Laboratories), which contains 3.1 mM KNO_3_ as well as all the necessary nutrients to sustain plant growth except for phosphorus. To the medium was added either 500 mg/L 2-(*N*-morpholino)­ethanesulfonic acid (final pH 5.7) (0 mM P Buffer), 1 or 0.5 mM KH_2_PO_4_ buffer (1 mM and 0.5 mM P Buffer, respectively), or a suspension of nanoparticles to provide a 1 or 0.5 mM phosphate concentration. Nanoparticles without phosphate were added to the media to provide the same weight of nanoparticles as the phosphate-containing ones. In both cases, the nanoparticles were first resuspended in 10 mL of MS medium followed by 20 min sonication, prior to obtaining the final dilution (1 or 0.5 mM phosphate concentration). Plants were irrigated once per week with 50 mL of MS medium provided from the top, containing 0, 0.5, or 1 mM P provided as KH_2_PO_4_ (positive control), an equivalent amount of TPP-containing lignin nanoparticles (1 mM or 0.5 mM P nanoparticles), or an equivalent amount of lignin nanoparticles that did not incorporate TPP (negative control). The growth chamber conditions were 22 °C and 60% relative humidity with a 16-h-light/8-h-dark photoperiod and 100 μE/m^2^/s of white light.

#### Shoot Weight and Phosphate Content

The shoots from 4-week-old plants were excised, and their fresh weight was determined. Phosphate quantification was performed as described by Ames et al.^60^ Briefly, shoot tissue was placed in pure water and subjected to three freeze–thaw cycles to release the inorganic phosphate, which was quantified by the molybdate method.^61^

#### Gene Expression Analysis

Total RNA was extracted from shoots using an RNA purification kit (Promega ReliaPrep RNA Tissue Miniprep System) as described by the manufacturer.^62^ One microgram of RNA was used for cDNA synthesis using the M-MLV Reverse Transcriptase (Promega) and oligo d­(T)­15, following the manufacturer’s instructions.^63^ RT-qPCR analysis was performed using SYBR Select Master Mix (Applied Biosystems) and ACT2 as the reference gene. The following forward and reverse primers were used for the PCR reactions: ACT2 (5′-CCGCTCTTTCTTTCCAAGC-3′ and 5′-CCGGTACCATTGTCACACAC-3′), MGD3 (5′-AGAGGCCGGTTTAATGGAG-3′ and 5′-CATCAGAGGATGCACGCTA-3′), IPS1 (5′-AGACTGCAGAAGGCTGATTCAGA-3′ and 5′-TTGCCCAATTTCTAGAGGGAGA-3′), and SPX1 (5′-TCCCTGCTAACGAAACTGAGT-3′ and 5′-AGAGGCGGCAATGAAAACAC-3′). For each analysis, samples were analyzed from 3 different plants, analyzing at least 2 shoots per plant.

### Procedures

#### Phenolation of Soda Lignin

The phenolation of lignin was performed using a modification of the procedure reported by Jiao et al.^64^ 5 g of soda lignin was dissolved in 10 g (0.106 mol) of phenol. Then, 2.5 mL H_2_SO_4_ were added, and the mixture was stirred at 110 °C for 30 min. After that, the mixture was slowly added to 300 mL of aqueous HCl solution (pH = 2) under continuous stirring to precipitate the phenolated lignin. Subsequently, the precipitate was filtered through a paper filter and washed three times with acidified water (pH = 3) and three times with deionized water. The dark brown powder was finally dried under vacuum at room temperature overnight to obtain ∼12 g of product. The ^1^H NMR spectrum of phenolated lignin is included in Supporting Information Figure S3B. GPC analysis provided *M*_n_ = 2300 g/mol, *M*_w_ = 3400 g/mol, and Đ = 1.50 (Supporting Information Table S1 and Supporting Information Figure S4). Phenolation of soda lignin resulted in an increase in the phenol group content from 0.42 to 1.79 mmol/g, as determined by ^31^P NMR spectroscopy (Supporting Information Figure S5 and Supporting Information Table S1).

#### Amination of Phenolated Lignin

The amination of the phenolated lignin was performed following a modification of the procedure by Jiao et al.^64^ 4 g of phenolated lignin was dissolved in 20 mL of 0.4 M NaOH by stirring for 15 min. After that, 20 mL of ethylene diamine (0.30 mol) and 20 mL of 37% aqueous formaldehyde solution (0.25 mol) were added, and the mixture was stirred for 3 h at 60 °C. The product was then dialyzed against deionized water using a dialysis tube with a molecular weight cutoff of 1 kDa (Dialysis Membrane Spectra/PorRTM 7, pore size 1000, 38 mm, Carl Roth GmbH & Co.) for 24 h and finally freeze-dried for 48 h. The final product amount was ∼2 g. The ^1^H NMR spectrum of aminated lignin is shown in Supporting Information Figure S3C. GPC analysis provided *M*_n_ = 2200 g/mol, *M*_w_ = 3400 g/mol, and Đ = 1.50 (Supporting Information Table S1 and Supporting Information Figure S4). Elemental analysis of the Mannich-modified lignin revealed a nitrogen content of 8.5% (Supporting Information Table S1).

#### Synthesis of Lignin/TPP Nanoparticles

Nanoparticles were prepared using a 1.25:1 N/P molar ratio. To this end, 100 mg of aminated lignin was dissolved overnight in 20 mL of aqueous HCl solution at pH 2 (aminated lignin concentration of 5 mg/mL) and then filtered through a 0.45 μm pore size syringe filter (Chromafil Xtra, Macherey-Nagel). In a separate flask, 60 mg (0.163 mmol) of TPP was dissolved overnight in 20 mL of Milli-Q water and then filtered through a 0.22 μm pore size syringe filter (Chromafil Xtra, Macherey-Nagel). After that, the TPP solution was added dropwise to the aminated lignin solution using a syringe pump (Ismatec, Thermo Fisher Scientific) at a flow rate of 0.2 mL/min with continuous stirring. After the addition was complete, the pH of the solution was measured and adjusted to pH 5 with 0.5 M NaOH. A 2 μL aliquot of 1 mM NaCl was added to preserve the ionic strength, and the mixture was stirred for 45 min at room temperature. The mixture was then sonicated using a tip sonicator (Ultrasonic Processors, Vibra-Cell, probe diameter 1.2 cm) at 50% amplitude for 10 min to form a nanosuspension. The mixture was kept in an ice bath during the sonication and for another 10 min after the completion of the sonication. For the preparation of ionically cross-linked nanoparticles, the sample was directly freeze-dried with 10 mg/mL of sucrose as cryoprotectant.

For the preparation of covalently cross-linked nanoparticles, 12 mg of EDC (0.0626 mmol; 0.4 mol equiv with regard to TPP) was added to the nanosuspension. The reaction mixture was stirred vigorously for 1 min and then allowed to stand for 1.5 h. After that, the suspension was dialyzed overnight against deionized water using a membrane with a cutoff of 10 kDa (Snakeskin Dialysis Tubing, Thermo Scientific Pierce) to remove 1-ethyl-3-(3-(dimethylamino)­propyl)­urea byproduct and finally freeze-dried with 10 mg/mL of sucrose as a cryoprotectant. The final product was a clear powder containing 20 wt % nanoparticles and 80 wt % sucrose.

#### Synthesis of Lignin Nanoparticles

Lignin nanoparticles that do not contain a TPP cross-linker, which were used as a control for the tests on *A. thaliana*, were prepared following a previously published procedure.^65^ Briefly, 1 mL of 0.25 M HCl was added to a solution of 4 wt % soda lignin in ethylene glycol at a rate of 0.04 mL/min under vigorous stirring. After 2 h of stirring, the mixture was sonicated for 30 min at a frequency of 30 kHz in a TPC-40 ultrasonic bath (Telsonic AG, Switzerland) and then dialyzed through a membrane with a cutoff of 10 kDa. The nanoparticles were finally freeze-dried. The prepared lignin nanoparticles had an average diameter of 110.8 ± 11.9 nm, as measured by AFM.^65^

#### Acid Phosphatase Activity Test

The acid phosphatase activity was tested following the supplier’s guidelines,^66^ at pH 5.7 and room temperature. A 90 mM citrate buffer was prepared by dissolving 2.57 g of sodium citrate dihydrate (8 mmol) and 240 mg of citric acid (1 mmol) in 100 mL of Milli-Q water. The pH was adjusted to 5.7 with 0.5 M NaOH. A 15.2 mM *p*-nitrophenyl phosphate disodium salt hexahydrate (pNPP) solution was prepared by dissolving 56.4 mg (0.152 mmol) of pNPP in 10 mL of citrate buffer. Enzyme solutions containing 0.15, 0.20, and 0.25 U/mL were prepared in citrate buffer. To determine the enzymatic activities, a test vial was prepared that contained 500 μL of citrate buffer, 500 μL of pNPP solution, and 100 μL of the respective enzyme solution, as well as a blank vial that contained 500 μL of citrate buffer and 500 μL of pNPP solution. After 10 min, 4 mL of 0.1 M NaOH was added to the test vial, and 4 mL of 0.1 M NaOH and 100 μL of the respective enzyme solution were added to the blank vial. The absorbance at 410 nm was measured with a spectrophotometer (PerkinElmer Lambda 365), using a cuvette with a 1 cm path length. The enzymatic activity was calculated using the following equation:

$$U/\mathrm{mL}=\frac{{(A}_{\mathrm{test}}-A_{\mathrm{blank}})\times 5.1}{10\times 18.3\times 0.1}$$

In this equation, 5.1 = total volume (in mL) of solution, 10 = time of assay (in minutes) as per the Unit definition, 18.3 = millimolar extinction coefficient of pNPP at 410 nm (in mL mmol^–1^ cm^–1^), and 0.1 = volume (in mL) of enzyme used. Per definition, one unit of enzyme will hydrolyze 1.0 μmole of pNPP per minute at pH 5.7 and room temperature. The obtained value was 0.192 ± 0.006 U/mg, expressed as the average of the values calculated for each enzyme concentration ± standard deviation.

#### Calibration of the Malachite Green Assay

The calibration of the malachite green assay was performed following the supplier’s guidelines^67^ using 2.5 mM MES buffer at pH 5.7 as the medium. A working reagent was prepared by mixing Reagent A (10 mM ammonium molybdate, 1 N HCl, and 3.4% ethanol) and Reagent B (malachite green oxalate salt) in a volume ratio of 100:1. Solutions containing different concentrations of phosphate standard were prepared in MES buffer, namely 0, 8, 16, 24, 32, 40, 48, and 56 μM. In each well of a 96-well plate, 80 μL of phosphate standard solution and 20 μL of working reagent were mixed and left for 30 min in ambient conditions for color development and stabilization. The absorbance at 620 nm was then measured with a plate reader (Infinite 200 PRO, Tecan, Lifesciences). The experiment was performed in triplicate.

#### Assessment of Acid Phosphatase-Mediated Nanoparticle Degradation and Phosphatase Release

For these analyses, 30 mg of freeze-dried nanoparticles (corresponding to 6 mg of nanoparticles and 24 mg of sucrose, because the total weight includes 20 wt % nanoparticles and 80 wt % sucrose) were dispersed in 29 mL of 2.5 mM MES buffer at pH 5.7 by 10 min sonication. Then, 1 mL of enzyme solution in MES buffer to reach an activity of 0, 0.1, 1, or 10 mU/mL was added to the nanoparticles, and the resulting mixture was inserted in a dialysis cassette with a cutoff of 10 kDa and 30 mL maximum volume (Slide-A-Lyzer 10K Dialysis Cassettes G2, Thermo Scientific). The dialysis cassette was inserted into a beaker containing 1 L of MES buffer. A magnetic stirrer was inserted inside the beaker as well to allow a homogeneous concentration inside the medium. At each time point, 100 μL was taken out from the dialysis cassette, diluted to 2 mL with MES buffer, sonicated for 10 min, and analyzed with DLS and AFM to study the change in nanoparticle size and shape. 80 μL was taken from the medium outside the dialysis cassette and transferred into a well plate together with 20 μL of a working solution of the malachite green assay. After 30 min of incubation at ambient conditions, the absorbance at 620 nm was measured with a plate reader to quantify the released phosphate concentration. The experiment was repeated in triplicate.

## Supplementary Material



## Data Availability

The source data of this study are available from the Zenodo repository at https://doi.org/10.5281/zenodo.19659130

## Notes

Additional nanoparticle characterization details; results from dynamic light scattering, GPC, and NMR analyses (PDF)
