# Supplementary material for: Plant-Growth Synchronized, Acid Phosphatase-Responsive Lignin-Based Controlled Release Phosphorus Nanofertilizers
Source: Biomacromolecules. 2026 Apr 27;27(5):3176–87. doi: 10.1021/acs.biomac.5c02594 (PMC13169352; doi:10.1021/acs.biomac.5c02594)
Supplement: Supplementary file 1 [file bm5c02594_si_001.pdf]

Supporting Information

Plant-Growth Synchronized, Acid Phosphatase-Responsive Lignin-Based Controlled Release Phosphorus Nanofertilizers

*Alice Boarino [1±], Nicola Carrara [1±], Joaquin Clua [2,3], Nick Zahnd [1], Yves Poirier [2], Harm-Anton Klok [1]**

[1] Institut des Matériaux and Institut des Sciences et Ingénierie Chimiques, Laboratoire des Polymères, École Polytechnique Fédérale de Lausanne (EPFL), Station 12, 1015 Lausanne, Switzerland

[2] Department of Plant Molecular Biology, University of Lausanne, 1015 Lausanne, Switzerland

[3] Instituto de Biología Molecular y Celular de Plantas, Consejo Superior de Investigaciones Científicas, Universitat Politècnica de València, 46011 Valencia, Spain

alice.boarino@epfl.ch, nicola.carrara@epfl.ch, jclua@ibmcp.upv.es, nick.zahnd@unifr.ch, yves.poirier@unil.ch, harm-anton.klok@epfl.ch

± These authors contributed equally

* Corresponding author

CORRESPONDING AUTHOR: Email: harm-anton.klok@epfl.ch; Phone: + 41 21 693 4866

Table S1. Characterization of Soda lignin, phenolated lignin, aminated lignin and TPP loaded nanoparticles: hydroxyl group concentration per gram of lignin as obtained from ^{31}P-NMR spectroscopy; C, H and N content determined by elemental analysis; P content quantified with ICP and molecular weights analyzed by GPC.

	**Soda lignin**	**Phenolated lignin**	**Aminated lignin**	**Covalently crosslinked lignin/TPP nanoparticles**
Aliphatic OH (mmol/g)	1.88	0.18	0.12	
Syringyl OH (mmol/g)	1.89	1.36	0.91	
Guaiacyl OH (mmol/g)	1.06	0.45	0.16	
p-hydroxyphenyl OH (mmol/g)	0.42	1.79	0.75	
COOH (mmol/g)	1.00	0.31	0.32	
C%wt	56.2 ± 1.2	67.5 ± 2.3	63.3 ± 1.8	43.8 ± 0.8
H%wt	5.3 ± 0.10	5.5 ± 0.43	6.3 ± 0.31	6.1 ± 0.48
N%wt	0.5 ± 0.03	0.4 ± 0.09	8.5 ± 0.12	6.3 ± 0.27
P%wt				11.13 ± 0.68
M_n (g/mol)	2000	2300	2200	
M_w (g/mol)	3800	3400	3400	
Đ (-)	1.80	1.50	1.50	

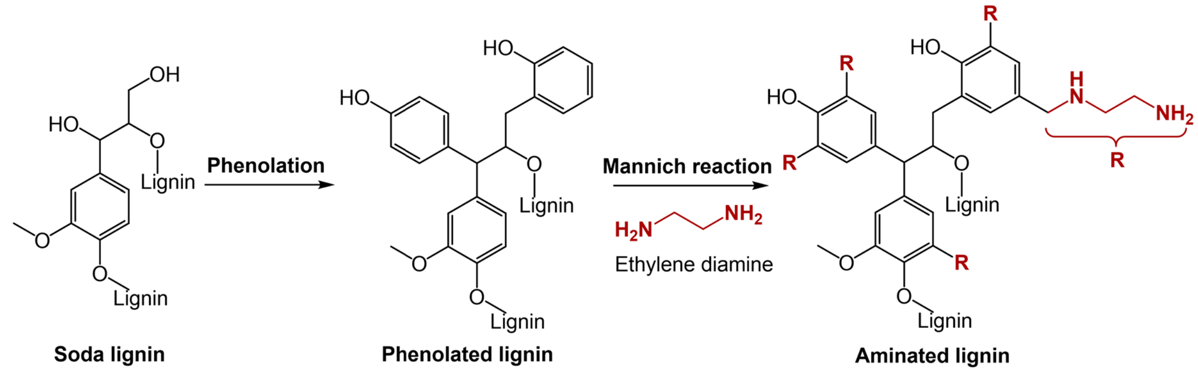

Scheme S1. Modification of Soda lignin via phenolation, and Mannich reaction.

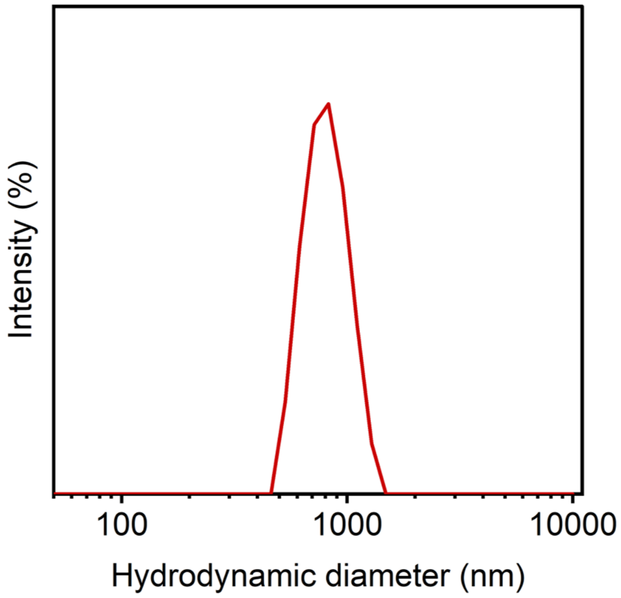

Figure S1. Particle size distribution of nanoparticles prepared by ionic gelation of aminated lignin with TPP.

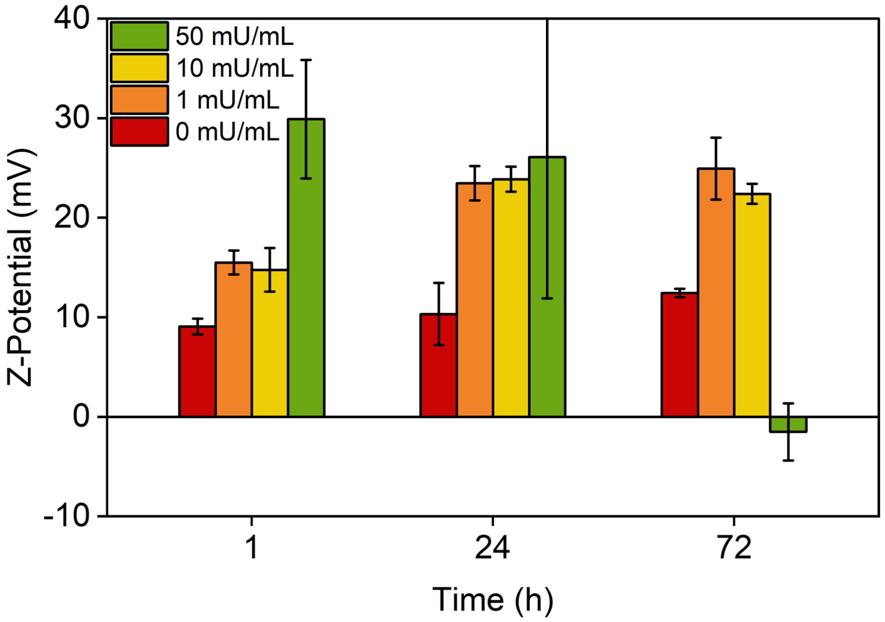

Figure S2. Zeta potentials of nanoparticles incubated with 0, 1, 10 and 50 mU/mL acid phosphatase, measured with DLS at 1 h, 24 h, and 72 h.

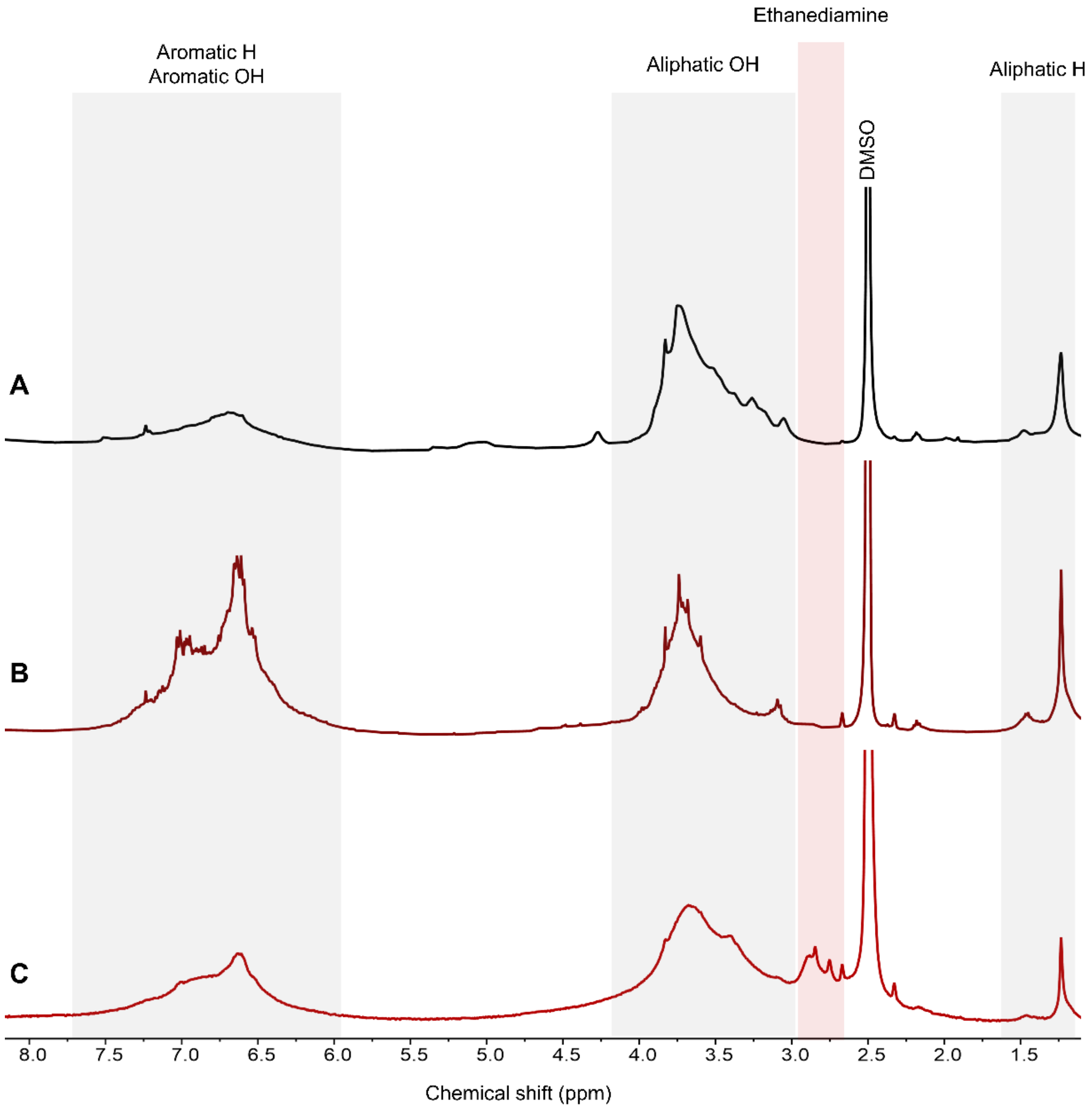

Figure S3. ^{1}H-NMR spectra (recorded in deuterated DMSO) of A) Soda lignin, B) phenolated lignin and C) aminated lignin.

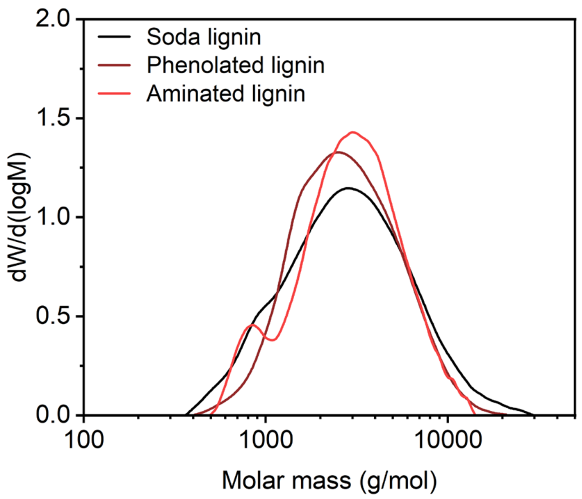

Figure S4. Molecular weight distributions of Soda lignin, phenolated lignin and aminated lignin.

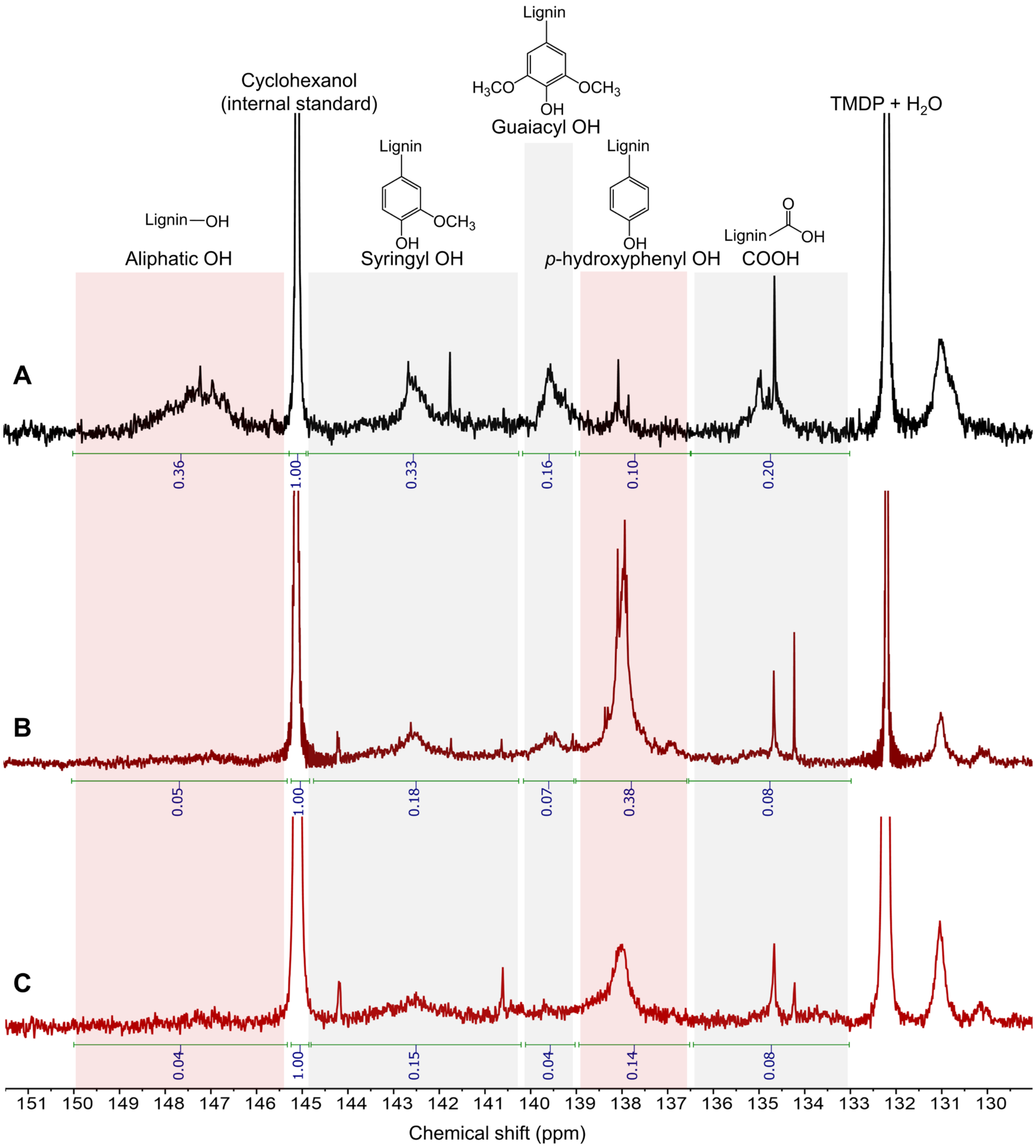

Figure S5. ^{31}P-NMR spectra of A) Soda lignin, B) phenolated lignin and C) aminated lignin.
